# Early childhood caries in Africa: prevalence, determinants, and implications for oral health promotion—an umbrella review

**DOI:** 10.3389/froh.2026.1901145

**Published:** 2026-06-25

**Authors:** Mohammed Farouk, Wahid Hafsa Oumayma, Oussama Bentahar

**Affiliations:** 1 Department of Odontology, Faculty of Medicine, Pharmacy and Dentistry, Sidi Mohammed Ben Abdellah University, Fez, Morocco; 2 Laboratory of Research in Health Sciences and Epidemiology, Faculty of Medicine, Pharmacy and Dentistry, Sidi Mohammed Ben Abdellah University, Fez, Morocco

**Keywords:** Africa, early childhood caries, oral health promotion, prevalence, prevention, risk factors, umbrella review

## Abstract

**Background:**

Early childhood caries (ECC) remains one of the most prevalent chronic diseases affecting children worldwide and represents a major public health challenge in many African settings. Understanding its determinants is essential for developing oral health promotion strategies and preventive policies aimed at improving child oral health and reducing inequalities.

**Methods:**

This umbrella review followed PRISMA guidelines and was registered in PROSPERO (CRD420261379153). Systematic reviews and meta-analyses published between January 2000 and May 2026 were identified through searches of MEDLINE, Scopus, Web of Science, and Embase. Methodological quality was assessed using AMSTAR 2, risk of bias using ROBIS, overlap using a citation matrix and corrected covered area (CCA), and certainty of evidence using a narrative GRADE approach. Findings were synthesized narratively.

**Results:**

Seven systematic reviews and meta-analyses were included. Methodological quality ranged from high to critically low, while ROBIS identified predominantly low overall risk of bias, although two reviews were judged to be at high risk of bias. The corrected covered area (CCA) was 4.7%, indicating a low degree of overlap among reviews. ECC prevalence ranged from 17% to 57%, with higher estimates reported in North and Southern Africa. The most consistently reported determinants were dietary, oral hygiene-related, sociodemographic, and breastfeeding and bottle-feeding-related factors, whereas maternal/caregiver-related, biological, health-related, and contextual determinants were supported by a smaller body of evidence. Certainty of evidence was moderate for ECC prevalence and the most consistently reported determinants, but low for maternal/caregiver-related, health-related, biological, and contextual determinants. These findings support evidence-informed oral health promotion and prevention strategies across African settings.

**Conclusion:**

ECC remains a substantial oral health burden in Africa and is influenced by interacting behavioral, sociodemographic, caregiver-related, biological, health-related, and healthcare access determinants. Comprehensive oral health promotion strategies should strengthen caregiver education, support healthy dietary and oral hygiene behaviors, improve access to preventive services, and integrate oral health into maternal and child health programs. Such approaches may help reduce ECC burden and oral health inequalities among vulnerable populations.

**Systematic Review Registration:**

PROSPERO, identifier CRD420261379153, http://www.crd.york.ac.uk/PROSPERO/view/CRD420261379153.

## Background

Early childhood caries (ECC) is defined as the presence of one or more decayed (non-cavitated or cavitated), missing (due to caries), or filled tooth surfaces in any primary tooth in children aged 71 months or younger, according to the American Academy of Pediatric Dentistry (AAPD) (1, 2). ECC is one of the most prevalent chronic conditions affecting young children worldwide and remains a major public health concern due to its early onset and potential long-term consequences on growth, development, and quality of life (1, 3).

The burden of ECC is disproportionately higher in low- and middle-income countries, including many African settings, where socioeconomic inequalities, limited access to preventive dental services, and ongoing nutritional and dietary transitions characterized by increased availability and consumption of processed foods, sugar-sweetened beverages, and energy-dense diets may increase exposure to ECC risk factors. In these contexts, ECC is often untreated, leading to pain, infection, impaired nutrition, and negative impacts on child development, as well as increased strain on already limited healthcare systems (3, 4). Beyond its clinical consequences, ECC contributes to health inequalities and may adversely affect children's well-being, educational participation, and quality of life (3, 5).

The etiology of ECC is multifactorial and involves a complex interaction between biological, behavioral, and environmental determinants. Key factors include frequent consumption of free sugars, inadequate oral hygiene practices, feeding behaviors such as prolonged or nocturnal bottle-feeding, and insufficient exposure to fluoride. In addition, broader contextual determinants—including socioeconomic status, caregiver knowledge, access to dental care, and environmental conditions—may influence both the development and progression of the disease. However, the relative contribution of these determinants appears to vary across populations and settings, reflecting differences in social and health system contexts (5, 6).

From an oral health promotion perspective, understanding the determinants of ECC is essential because many of these factors are potentially modifiable through preventive interventions, caregiver education, community-based programs, and public health policies. Early-life prevention strategies targeting dietary habits, oral hygiene practices, caregiver behaviors, and access to preventive services have the potential to reduce disease burden and improve oral health outcomes throughout the life course. Identifying the most consistently reported determinants may therefore help guide evidence-based prevention strategies and support efforts to reduce oral health disparities among children.

In recent years, several systematic reviews and meta-analyses have investigated the prevalence and determinants of ECC in different African regions. While these studies provide valuable insights, the available evidence remains fragmented and heterogeneous, with variations in methodological approaches, study populations, and reported outcomes limiting direct comparability and synthesis. Furthermore, most reviews have focused on specific countries or subregions, with limited efforts to integrate findings at a broader continental level.

Although several systematic reviews and meta-analyses have examined ECC prevalence and associated risk factors in African settings, the translation of this evidence into effective prevention strategies and oral health promotion initiatives remains challenging. Differences in methodological quality, regional coverage, and reported determinants make it difficult to identify the factors that are most consistently associated with ECC and most relevant for preventive action. Importantly, no previous umbrella review has comprehensively synthesized this body of evidence while simultaneously evaluating methodological quality, risk of bias, overlap between reviews, and certainty of evidence. Given the growing recognition of oral health as an integral component of overall child health, understanding the determinants of ECC is also essential for informing oral health promotion programs, reducing oral health inequalities, and improving equitable access to preventive services. These objectives are aligned with contemporary oral health promotion frameworks and with global efforts to achieve Sustainable Development Goal 3 (Good Health and Well-being) and Sustainable Development Goal 10 (Reduced Inequalities). This is particularly relevant in African settings where vulnerable and underserved populations often experience disproportionate disease burden and limited access to oral healthcare. Therefore, the present umbrella review aimed to synthesize the available evidence regarding the prevalence of ECC and its associated risk factors across African settings. Specifically, this review sought to determine the reported prevalence of ECC in African populations and to identify the risk factors that are most consistently associated with ECC across the available systematic reviews and meta-analyses, with the ultimate goal of informing oral health promotion strategies, preventive interventions, and public health decision-making.

## Methods

### Study design and registration

This umbrella review was conducted following The PRISMA 2020 guidelines (the Preferred Reporting Items for Systematic Reviews and Meta-Analyses) (7), in accordance with a validated step-by-step guide for umbrella reviews (8). It was registered in PROSPERO on 25 April 2026 (registration number: CRD420261379153). The review aimed to synthesize evidence from systematic reviews and meta-analyses on two key aspects: the prevalence of early childhood caries and its associated risk factors in Africa. Given the heterogeneity in review objectives, populations, diagnostic criteria, prevalence estimates, and reported determinants, findings were synthesized narratively rather than quantitatively pooled.

### Research question

The research question was formulated using the CoCoPop (Condition, Context, Population) framework.

The condition of interest was early childhood caries (ECC), defined as dental caries affecting the primary dentition in children under six years of age.

The context focused on prevalence estimates and associated risk factors in African settings.

The population comprised children residing in African countries within the early childhood age range. CoCoPop was selected because the primary objective of the review was to synthesize prevalence estimates and associated determinants of ECC rather than evaluate the effectiveness of an intervention or compare predefined exposure groups.

By restricting the condition to ECC, the present umbrella review ensures a biologically and developmentally homogeneous outcome, thereby enhancing the comparability and interpretability of the findings across included studies.

### Eligibility criteria

Studies were included if they were systematic reviews or meta-analyses focusing on early childhood caries (ECC) in pediatric populations residing in African countries. ECC was defined as dental caries affecting the primary dentition in children under six years of age. Eligible studies were required to report prevalence estimates and/or associated risk factors and to be available in full text and published in English between January 2000 and May 2026.

Studies were excluded if they were scoping reviews, narrative reviews, expert opinions, editorials, letters, conference abstracts, or primary studies; if they did not report extractable data on ECC prevalence or associated risk factors in African populations; if they focused exclusively on older children or permanent dentition without providing separate data for ECC; if they assessed individuals of African origin living outside Africa; or if the full text was unavailable.

### Search strategy

A systematic search was conducted in MEDLINE (via PubMed), Scopus, Web of Science, and Embase, restricted to English-language publications between January 2000 and May 2026. The final literature search was conducted on 10 May 2026.

The search strategy combined controlled vocabulary terms (MeSH, where applicable) and free-text keywords related to early childhood caries, prevalence, risk factors, determinants, children, preschool populations, Africa, and the names of all African countries. Relevant MeSH terms included “Dental Caries”, “Child, Preschool”, “Risk Factors”, and “Africa”, combined with free-text terms such as *early childhood caries, ECC, prevalence, epidemiology, and determinants*. The search strategy also incorporated the names of all African countries to maximize the identification of region-specific evidence. Equivalent database-specific subject headings, search fields, and keywords were adapted for Scopus, Web of Science, and Embase. Boolean operators (AND/OR) were used to optimize retrieval. The search strategy was informed by previously published systematic reviews on early childhood caries epidemiology in African populations (2, 3) and was iteratively refined to maximize sensitivity and specificity.

To improve transparency and methodological rigor, the search strategy was reported in accordance with the Peer Review of Electronic Search Strategies (PRESS) guideline (9). Filters for systematic reviews and meta-analyses were applied in all databases. Detailed database-specific search strategies, including complete search syntaxes and database-specific search yields for PubMed, Scopus, Web of Science, and Embase, are provided in Supplementary Appendix 1.

### Study selection

Retrieved records were imported into EndNote for duplicate removal. Two reviewers (F.M. and H.W.) independently screened titles and abstracts, followed by full-text review of eligible studies. Disagreements were resolved by consultation with a third reviewer (B.O.).

### Characteristics of included studies

Characteristics of the included reviews were extracted independently by two reviewers (F.M. and W.H.) using a standardized data extraction form. Extracted information included the first author, year of publication, type of review, databases searched, review objectives, geographical setting, and population characteristics. Any discrepancies were resolved through discussion and consensus, with consultation of a third reviewer (B.O.) when necessary.

### Assessment of overlap among included reviews

Overlap among included reviews was assessed quantitatively using a citation matrix, in which primary studies included in each systematic review were mapped across reviews. Two reviewers (F.M. and H.W.) independently assessed the overlap of primary studies across the included reviews using a citation matrix and calculated the corrected covered area (CCA), with discrepancies resolved by discussion or consultation with a third reviewer (B.O.). The degree of overlap was quantified using the corrected covered area (CCA), calculated as follows: CCA = (N−r)/((r × c)−r). Where N represents the total number of included studies across all reviews (counting repeated occurrences), r is the number of unique primary studies, and c is the number of reviews. The resulting CCA value was interpreted using established thresholds, with 0%–5% indicating low overlap, 6%–10% moderate overlap, 11%–15% high overlap, and >15% very high overlap (10). This approach allowed systematic identification of shared primary studies across reviews and provided a quantitative measure of overlap to support interpretation of the synthesized evidence.

### Quality assessment

The methodological quality of the included reviews was assessed using AMSTAR 2 (A Measurement Tool to Assess Systematic Reviews), a validated critical appraisal tool designed for systematic reviews of randomized and non-randomized studies of healthcare interventions. Two reviewers (F.M. and H.W.) independently performed the methodological quality assessment of the included studies using the AMSTAR 2 tool. Any discrepancies between reviewers were resolved through discussion, and when consensus could not be reached, a third reviewer (B.O.) was consulted to adjudicate.

The AMSTAR 2 instrument, as described by Shea et al. (11), was used to classify reviews as high, moderate, low, or critically low quality based on the number and nature of critical and non-critical weaknesses.

### Risk of bias assessment

The risk of bias of the included systematic reviews was assessed using the Risk of Bias in Systematic Reviews (ROBIS) tool (12). ROBIS evaluates the review process across four domains: study eligibility criteria, identification and selection of studies, data collection and appraisal, and synthesis and findings. Assessments were performed independently by two reviewers (F.M. and W.H.). Any disagreements were resolved through discussion and consensus, with consultation of a third reviewer (B.O.) when necessary. Based on ROBIS guidance, each review was classified as having a low, high, or unclear overall risk of bias.

### Certainty of evidence assessment

The certainty of evidence was assessed using a narrative GRADE approach adapted for umbrella reviews (13). Assessments were performed independently by two reviewers (F.M. and W.H.). Any disagreements were resolved through discussion and consensus, with consultation of a third reviewer (B.O.) when necessary.

Certainty assessments were conducted at the level of determinant categories rather than individual risk factors. Certainty judgments considered methodological quality (AMSTAR 2), overall ROBIS risk-of-bias judgments, consistency of findings across reviews, indirectness, imprecision, publication bias, and overlap between reviews (CCA).

Given the absence of sufficiently comparable pooled effect estimates and the substantial heterogeneity across included reviews, certainty ratings were derived using a structured narrative application of GRADE domains rather than a formal quantitative GRADE assessment.

Because certainty assessments were conducted narratively, overall certainty ratings reflected the collective consideration of all GRADE domains and methodological indicators rather than a predefined quantitative downgrading algorithm.

For each determinant category, judgments for risk of bias, inconsistency, indirectness, and imprecision were classified as either “not serious” or “serious” according to predefined criteria. Risk of bias was considered serious when the available evidence was derived predominantly from reviews rated as low or critically low quality according to AMSTAR 2 or judged as having a high overall risk of bias according to ROBIS. Inconsistency was considered serious when substantial variation in findings, direction of associations, or prevalence estimates was observed across reviews and could not be adequately explained by differences in populations, settings, or review methodologies. Indirectness was considered serious when evidence was limited to specific populations, regions, or contexts that reduced the applicability of findings to African settings as a whole. Imprecision was considered serious when determinant categories were supported by a limited number of reviews, sparse evidence, or insufficiently robust data to support stable conclusions. Publication bias was considered not assessable because most included reviews did not provide sufficient information to formally evaluate publication bias across determinant categories.

Evidence was downgraded when one or more GRADE domains were judged serious. When heterogeneity was primarily attributable to differences in geographical settings, populations, study characteristics, or review methodologies rather than conflicting directions of association, inconsistency was not automatically considered serious. Certainty judgments also incorporated the degree of overlap between reviews, with lower overlap supporting greater confidence in the independence of the evidence base. Because most included reviews synthesized predominantly observational evidence and reported heterogeneous outcomes, no outcome was upgraded beyond moderate certainty.

## Data extraction

Data were independently extracted by two investigators (F.M. and H.W.) using a standardized Microsoft Excel-based data extraction form developed *a priori* for this review. Disagreements were resolved through discussion, and when consensus could not be reached, a third reviewer (B.O.) was consulted. Extracted items included: first author's name, year of publication, type of review, population characteristics (age range within early childhood), country or region, reported prevalence estimates and pooled prevalence values of early childhood caries (ECC), and associated risk factors.

Given the methodological and clinical heterogeneity across the included reviews, particularly in study settings, diagnostic criteria, and reported outcomes, no quantitative meta-analysis was performed. Instead, findings were synthesized narratively.

## Results

### Study selection

**Figure 1:**
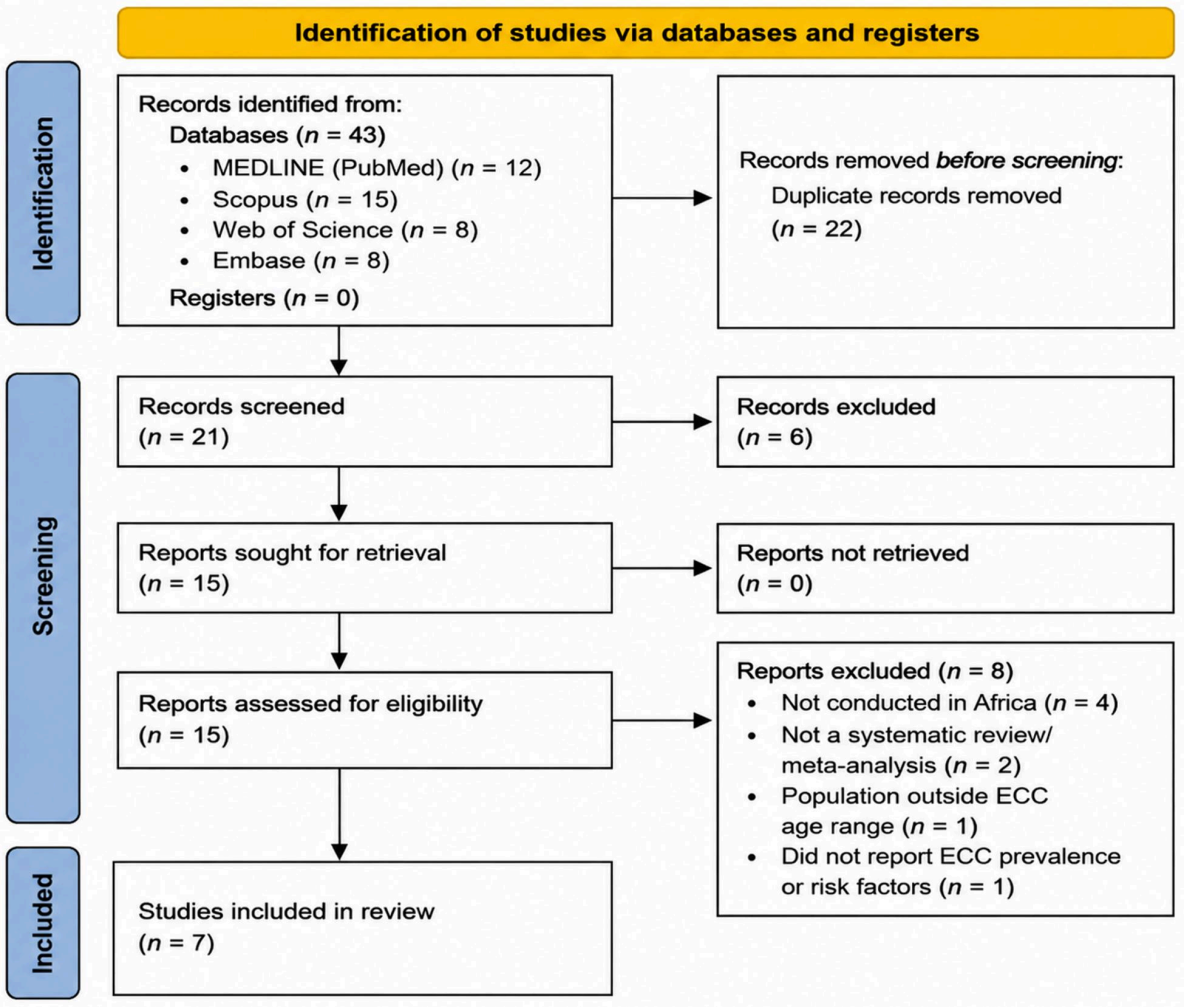
PRISMA 2020 flow diagram.

As illustrated in the flow diagram (Figure 1), 43 records were retrieved from the database searches. After removal of 22 duplicates, 21 records underwent title and abstract screening, of which 6 were excluded due to irrelevant themes. Full-text review was performed on 15 records, and 8 were excluded for not meeting the eligibility criteria, including non-eligible study designs and studies not specifically addressing early childhood caries. A total of 7 systematic reviews and meta-analyses were ultimately included in the final synthesis. These reviews met all predefined eligibility criteria and provided evidence on ECC prevalence and/or associated risk factors in African pediatric populations.

The study selection process was conducted independently by two reviewers, with excellent inter-reviewer agreement (*κ* = 0.82).

### Characteristics of included studies

The seven included reviews were published between 2022 and 2026 and covered multiple African settings, including South Africa (2, 16), Nigeria (17, 19), North Africa (14), Southern Africa (18), and continental African populations (15). Three reviews focused exclusively on ECC prevalence (2, 15, 17), one on associated risk factors (16), and three on both prevalence and determinants (14, 18, 19). Four reviews included meta-analyses (14, 17–19), whereas three provided narrative syntheses (2, 15, 16). Detailed characteristics of the included reviews are presented in (Table 1). Inter-reviewer agreement was excellent (*κ* = 0.85).

**Table 1:** Characteristics of included studies.

Included review	Year	Type of review	Databases	Review objective	Country/Region	Population
Kimmie-Dhansay F et al. (2)	2025	Systematic review	Scopus, EBSCO, Science Direct, PubMed, African Journal Online	Prevalence only	South Africa	0–6 years
Kimmie-Dhansay F et al. (15)	2026	Systematic review	EBSCO, PubMed, Science Direct, Google Scholar, Scopus, AFRICA-WIDE INFO	Prevalence only	Africa (13 countries)	0–6 years
Okolo CC et al. (17)	2024	Systematic review and meta-analysis	PubMed, Scopus, EBSCOhost, African Journal Online (AJOL)	Prevalence only	Nigeria	0–6 years
Kimmie-Dhansay F et al. (16)	2022	Systematic review	Scopus, Embase, PubMed, Science Direct	Risk factors only	South Africa	0–6 years
Famurewa BA et al. (14)	2025	Systematic review and meta-analysis	PubMed, Scopus, Web of Science, Embase, African Journals Online	Prevalence and risk factors	North Africa	0–5 years
Ogunlade OB et al. (18)	2026	Systematic review and meta-analysis	PubMed, Scopus, Web of Science, AJOL	Prevalence and risk factors	Southern Africa	0–6 years
Orhorhoro EE et al. (19)	2026	Systematic review and meta-analysis	PubMed, Web of Science, African Journals Online (AJOL), Scopus, ProQuest, Directory of Open Access Journals (DOAJ), Grey literature	Prevalence and risk factors	Nigeria	0–71 months (0–6) years

### Assessment of overlap among included reviews

Inter-reviewer agreement was excellent (*κ* = 0.87). Pairwise overlap ranged from 0 to 23 shared primary studies, with the highest overlap observed between the Nigerian reviews by Okolo et al. (17) and Orhorhoro et al. (19). The corrected covered area (CCA) was 4.7%, indicating a low degree of overlap and minimal risk of double-counting across the included reviews. Detailed overlap results are presented in (Table 2).

**Table 2:** Pairwise overlap (number of shared primary studies).

Review	Kimmie 2022 (2)	Kimmie 2022 (16)	Famurewa 2025 (14)	Okolo 2024 (17)	Ogunlade 2026 (18)	Kimmie 2026 (15)	Orhorhoro EE et al. (19)
Kimmie 2022 (2)	—	5	0	0	6	9	1
Kimmie 2022 (16)	5	—	0	0	4	7	1
Famurewa 2025 (14)	0	0	—	0	0	5	0
Okolo 2024 (17)	0	0	0	—	6	8	23
Ogunlade 2026 (18)	6	4	0	6	—	7	1
Kimmie 2026 (15)	9	7	5	8	7	—	2
Orhorhoro EE et al. (19)	1	1	0	23	1	2	—

### Quality assessment

Methodological quality ranged from high to critically low according to AMSTAR 2. Two reviews (14, 19) were rated as high quality, three reviews (2, 15, 18) as moderate quality, one review (17) as low quality, and one review (16) as critically low quality. Overall, most evidence was derived from reviews of moderate-to-high methodological quality. Inter-reviewer agreement was substantial (*κ* = 0.79). Detailed results are presented in (Table 3).

**Table 3:** Quality assessment of included systematic reviews using AMSTAR 2**.**

Authors	Famurewa et al. 2025 (14)	Kimmie et al. 2022 (16)	Kimmie et al. 2025 (2)	Kimmie et al. 2026 (15)	Okolo et al. 2024 (17)	Ogunlade et al. 2026 (18)	Orhorhoro EE et al. 2026 (19)
AMSTAR2 Items							
1. Did the research questions and inclusion criteria for the review include the components of PICO?	Yes	No	No	No	Yes	No	Yes
2. Did the report of the review contain an explicit statement that the review methods were established prior to the conduct of the review and did the report justify any significant deviations from the protocol?	Yes	No	P Yes	Yes	No	Yes	Yes
3. Did the review authors explain their selection of the study designs for inclusion in the review?	Yes	Yes	Yes	Yes	Yes	Yes	Yes
4. Did the review authors use a comprehensive literature search strategy?	Yes	Yes	Yes	Yes	Yes	Yes	Yes
5. Did the review authors perform study selection in duplicate?	Yes	No	Yes	No	Yes	Yes	Yes
6. Did the review authors perform data extraction in duplicate?	Yes	No	Yes	Yes	Yes	Yes	Yes
7. Did the review authors provide a list of excluded studies and justify the exclusions?	P Yes	P Yes	P Yes	Yes	P Yes	Yes	Yes
8. Did the review authors describe the included studies in adequate detail?	Yes	Yes	Yes	Yes	Yes	Yes	Yes
9. Did the review authors use a satisfactory technique for assessing the risk of bias (RoB) individual studies that were included in the review?	Yes	Yes	Yes	Yes	Yes	Yes	Yes
10. Did the review authors report on the sources of funding for the studies included in the review?	No	No	No	No	No	No	No
11. If a meta-analysis was performed, did the review authors use appropriate methods for statistical combination of results?	Yes	NA	NA	Yes	Yes	Yes	Yes
12. If a meta-analysis was performed, did the review authors assess the potential impact of RoB in individual studies on the results of the meta-analysis or other evidence synthesis?	Yes	NA	NA	Yes	Yes	Yes	Yes
13. Did the review authors account for RoB in individual studies when interpreting/discussing the results of the review?	Yes	No	Yes	Yes	Yes	Yes	Yes
14. Did the review authors provide a satisfactory explanation for, and discussion of, any heterogeneity observed in the results of the review?	Yes	No	Yes	Yes	Yes	Yes	Yes
15. If the review authors performed quantitative synthesis, did they carry out an adequate investigation of publication bias (small study bias) and discuss its likely impact on the results of the review?	Yes	NA	Yes	Yes	Yes	Yes	Yes
16. Did the review authors report any potential sources of conflict of interest, including any funding they received for conducting the review?	Yes	Yes	Yes	Yes	Yes	Yes	Yes
No. of critical domains weaknesses (negatively answered)	0	2	0	0	1	0	0
Final rating	High	Critically low	Moderate	Moderate	Low	Moderate	High

AMSTAR 2 items assessed: 1, PICO description; 2, protocol registration before the conduct of the review; 3, study design included in the review; 4, comprehensive search strategy; 5, two authors study selection; 6, two authors’ study extraction; 7. justification for excluding individual studies; 8, included studies described in detail; 9, risk of bias for the primary studies being included in the review; 10, source of funding of primary studies; 11, appropriate method of meta-analysis; 12, impact of the risk of bias of primary studies on the results of the meta-analysis; 13, risk of bias consideration when discussing the results of the review; 14, explaining and discussing the heterogeneity; 15, assessing the presence and likely impact of publication bias; 16, conflicts of interest and funding sources declared.

High: 0-1 noncritical weakness. Moderate: > 1 noncritical weakness. The systematic review has more than one weakness, but no critical flaws. Low: 1 critical flaw with or without noncritical weaknesses. Critically low: > 1 critical flaw with or without noncritical weaknesses.

**Key:**

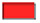
 no.

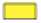
 partial yes.

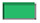
 yes.

N/A, not applicable.

### Risk of bias assessment

Inter-reviewer agreement was substantial (*κ* = 0.78). Five reviews were judged to have a low overall risk of bias (2, 14, 15, 18, 19), whereas two reviews were classified as high risk of bias (16, 17). No review received an unclear ROBIS judgment. Detailed ROBIS assessments are presented in (Table 4).

**Table 4:** ROBIS assessment of included systematic reviews.

Review	Domain 1: Study eligibility criteria	Domain 2: Identification and selection of studies	Domain 3: Data collection and study appraisal	Domain 4: Synthesis and findings	Overall ROBIS judgment
Famurewa et al., 2025 (14)	Low concern	Low concern	Low concern	Low concern	Low risk
Kimmie-Dhansay et al., 2022 (16)	High concern	High concern	High concern	High concern	High risk
Kimmie-Dhansay et al., 2025 (2)	Low concern	Low concern	Low concern	Low concern	Low risk
Kimmie-Dhansay et al., 2026 (15)	Low concern	Low concern	Low concern	Low concern	Low risk
Okolo et al., 2024 (17)	Low concern	High concern	High concern	High concern	High risk
Ogunlade et al., 2026 (18)	Low concern	Low concern	High concern	Low concern	Low risk
Orhorhoro EE et al. (19)	Low concern	Low concern	Low concern	Low concern	Low risk

### Certainty of evidence

Inter-reviewer agreement was substantial (*κ* = 0.77). Moderate-certainty evidence supported ECC prevalence and the most consistently reported determinant categories, including dietary (14, 16, 18, 19), oral hygiene-related (14, 16, 18, 19), sociodemographic (14, 16, 18, 19), and breastfeeding and bottle-feeding-related determinants (14, 16, 18, 19). Maternal/caregiver-related (16, 19), biological (16, 19), health-related (16, 19), and contextual determinants (16, 18, 19) were supported by low-certainty evidence. Detailed GRADE assessments are presented in (Table 5).

**Table 5:** GRADE assessment of certainty of evidence.

Outcome	No. of Reviews	Risk of Bias	Inconsistency	Indirectness	Imprecision	Publication Bias	GRADE Rating
ECC prevalence	5	Not serious	Serious	Not serious	Not serious	Not assessable	⊕⊕⊕○ Moderate
Dietary factors	4	Not serious	Not serious	Not serious	Serious	Not assessable	⊕⊕⊕○ Moderate
Oral hygiene factors	4	Not serious	Not serious	Not serious	Serious	Not assessable	⊕⊕⊕○ Moderate
Sociodemographic factors	4	Not serious	Not serious	Not serious	Serious	Not assessable	⊕⊕⊕○ Moderate
Breastfeeding and bottle-feeding risk factors	4	Not serious	Serious	Not serious	Not serious	Not assessable	⊕⊕⊕○ Moderate
Maternal/Caregiver-related factors	2	Serious	Not serious	Serious	Not serious	Not assessable	⊕⊕○○ Low
Biological factors	2	Serious	Not serious	Serious	Not serious	Not assessable	⊕⊕○○ Low
Health related factors	2	Serious	Not serious	Not serious	Serious	Not assessable	⊕⊕○○ Low
Contextual factors	3	Not serious	Serious	Serious	Not serious	Not assessable	⊕⊕○○ Low

### Prevalence of early childhood caries

Across the included reviews, ECC prevalence ranged from 17% to 57% (Table 6). The highest prevalence was reported in North Africa by Famurewa et al. (14) (57%), followed by Southern Africa in Ogunlade et al. (18) (52%). Continental estimates reported by Kimmie-Dhansay et al. (15) reached 37.9%, whereas South African reviews reported a prevalence of 44.9% (2). Lower prevalence estimates were reported in Nigeria, ranging from 17% in Okolo et al. (17) to 22% in Orhorhoro et al. (19). Overall, all included reviews indicate that ECC remains a substantial oral health burden among African children.

**Table 6:** Prevalence of early childhood caries reported in included studies.

Author/year	Type	Country	Population	Prevalence
Famurewa et al. 2025 (14)	Systematic review and meta-analysis	North Africa	0–5 years	57%
Kimmie et al. 2022 (16)	Systematic review	South Africa	0–6 years	No pooled prevalence estimate reported
Kimmie et al. 2026 (15)	Systematic review	Africa (13 countries)	0–6 years	37.9%
Kimmie et al. 2022 (2)	Systematic review	South Africa	0–6 years	44.94%
Okolo et al. 2024 (17)	Systematic review and meta-analysis	Nigeria	0–6 years	17%
Ogunlade et al. 2026 (18)	Systematic review and meta-analysis	Southern Africa	0–6 years	52%
Orhorhoro EE et al. (19)	Systematic review and meta-analysis	Nigeria	0–6 years	22%

### Risk factors

Risk factors reported across the included reviews were grouped into eight domains: sociodemographic, dietary, oral hygiene-related, biological, breastfeeding and bottle-feeding-related, maternal/caregiver-related, health-related, and contextual factors (Table 7).

**Table 7:** Risk factors reported in included studies.

Category	Risk factor	Reference
Sociodemographic factors	Age	(14, 16, 19)
	Male gender	(14, 19)
	Socioeconomic status	(16, 18, 19)
	Residential location	(16, 18)
	Presence of siblings	(14)
	Parental education	(16, 18, 19)
	Peri-urban school	(18)
	Limited access to dental services	(18)
	Unemployed or middle-income parents	(18)
	Coloured ethnicity	(18)
	Older preschool children (4–5 years)	(18)
	Birth rank (first, only, last child)	(19)
	Private or Public school	(19)
Dietary factors	Frequent intake of sugary foods	(14, 19)
	Nocturnal feeding	(14, 19)
	Feeding on demand	(14)
	Frequency of sugar intake per day	(16)
	Maternal sugar intake frequency	(16)
	Calorie intake	(16)
	Macronutrient intake	(16)
	Micronutrient intake	(16)
	Wasting and stunting	(16)
	Mode of sugar intake	(16)
	Reasons for giving sugar	(16)
	Age at the introduction to SSB and sugar-sweetened food	(16)
	Added sugar	(16)
	Total sucrose intake	(16)
	Knowledge caries potential sugar	(16)
	Fibre intake	(16)
	Carbohydrate intake	(16)
	Higher household sugar expenditure (urban)	(18)
	Daily consumption of candies/sugary drinks	(18)
	High sugar exposure and low fluoride (urban children)	(18)
	Improper feeding habits (bottle use, sugary diets)	(18)
	School time snacking	(19)
Oral hygiene related factors	Poorer oral hygiene status	(18, 19)
	Once daily toothbrushing	(18)
	Brushing frequency and timing	(14, 16)
	History and timing of dental visits	(14)
	Brushing method	(14)
	Parental supervision	(14, 18)
	Lack of OH instructions	(16)
	Knowledge of dental plaque	(16)
	Parents knowledge of the importance of primary teeth	(16)
	Knowledge of when to start brushing teeth	(16)
	Children cleaned teeth by themselves	(16)
	Use of toothbrush or cloth	(16)
	Use toothpaste when brushing	(16)
	Parental brushing of child's teeth	(16)
	Knowledge of oral health	(16)
	Delayed brushing	(16, 18)
	Higher plaque levels	(16)
	Delayed brushing initiation (> 12 months)	(18)
	Limited caregiver knowledge of toothbrushing	(18)
	Use of glycerine for cleaning	(19)
Biological factors	Level of plaque present	(16)
	Lactobacillus presence	(16)
	Streptococcus mutans presence	(16)
	Salivary flow rate	(16)
	Salivary buffering capacity	(16)
	Veillonella/Actinomyces presence	(16)
	Tooth anatomy	(19)
	Primary molar	(19)
Breastfeeding and bottle-feeding factors	Sweetened infant beverages	(16)
	Contents of bottle	(16)
	Mechanism of bottle/breast feeding	(16)
	Length of Breastfeeding	(16)
	Knowledge that frequent and prolonged bottle-feeding can cause dental caries	(16)
	Put child to sleep with a bottle	(16)
	Mode of feeding breast/bottle	(16)
	Age at weaning from breastfeeding or bottle feeding	(14)
	Breastfeeding beyond 18 months	(18, 19)
	Nighttime feeding > 2 times/night	(18)
	Exclusive breastfeeding and nursing at the breast	(19)
Maternal/Caregiver factors	Poor knowledge of oral health	(16, 19)
	Maternal prenatal medical history	(16)
	Knowledge of the presence of primary teeth important	(16)
	Parents examine child's teeth daily	(16)
	Parents’ knowledge of sugar in medication	(16)
	Prenatal supplementation	(16)
	Maternal premastication of food	(19)
	Maternal age	(19)
Health related factors	HIV infection	(19)
	Low CD4	(19)
	Sickle cell anemia	(19)
	Nutritional extremes (underweight, overweight)	(19)
	Perinatal complications	(16)
Contextual factors	Distance to nearest oral health facility	(16)
	Fluoride levels	(16, 18)
	Fluoride supplementation	(16)
	Reason for dental visit	(16)
	Prior dental visit	(19)

Dietary determinants were consistently reported across four reviews (14, 16, 18, 19) and included frequent sugar consumption, nocturnal feeding, feeding on demand, and school-time snacking. Oral hygiene-related determinants were also reported across four reviews (14, 16, 18, 19), with poor oral hygiene, delayed toothbrushing initiation, inadequate parental supervision, and limited caregiver oral health knowledge identified as common risk factors.

Sociodemographic determinants were reported across four reviews (14, 16, 18, 19) and included socioeconomic disadvantage, lower parental educational attainment, residential environment, and limited access to dental services. Breastfeeding and bottle-feeding-related determinants were similarly identified across four reviews (14, 16, 18, 19), particularly prolonged breastfeeding, nighttime feeding, bottle-feeding practices, and delayed weaning.

Biological determinants (16, 19), maternal/caregiver-related determinants (16, 19), health-related determinants (16, 19), and contextual determinants (16, 18, 19) were reported less frequently and were supported by a smaller body of evidence. Detailed determinants identified within each category are presented in (Table 7).

## Discussion

### Principal findings

This umbrella review provides a comprehensive synthesis of the available evidence on the prevalence and determinants of early childhood caries (ECC) across African settings. By restricting inclusion to systematic reviews and meta-analyses focusing exclusively on ECC in children under six years of age, the review offers a focused overview of a major oral health challenge affecting young children across the continent. The incorporation of methodological quality assessment (AMSTAR 2), risk of bias evaluation (ROBIS), overlap analysis using the corrected covered area (CCA), and certainty assessment through a narrative GRADE approach strengthened the robustness and interpretability of the synthesized evidence.

Overall, seven systematic reviews and meta-analyses were included, covering evidence from multiple African countries and regions. ECC prevalence ranged from 17% to 57%, with higher estimates reported in North and Southern Africa and lower estimates observed in Nigerian populations. Despite this variability, all included reviews consistently identified ECC as a substantial oral health burden among African children. The observed differences likely reflect variations in sociodemographic conditions, dietary environments, oral hygiene practices, access to preventive services, fluoride exposure, and healthcare system characteristics across settings.

The most consistently reported determinants were dietary factors, oral hygiene-related factors, sociodemographic factors, and breastfeeding and bottle-feeding-related factors. These determinants were supported by moderate-certainty evidence and were reported across multiple reviews. In contrast, biological, maternal/caregiver-related, health-related, and contextual determinants were supported by a smaller body of evidence and generally demonstrated lower certainty. Importantly, the low corrected covered area (CCA = 4.7%) indicates minimal overlap among included reviews, suggesting that the identified patterns were derived largely from independent bodies of evidence rather than repeated inclusion of the same primary studies. Furthermore, ROBIS assessments indicated that most included reviews were at low risk of bias, whereas only two reviews were judged to be at high risk of bias. Narrative GRADE assessments supported moderate certainty for ECC prevalence and the most consistently reported determinant categories. Collectively, these findings provide a reasonably robust evidence base for informing oral health promotion strategies and preventive interventions targeting ECC across African settings.

The determinants identified across the included reviews can be broadly organized into three interrelated levels. First, behavioral and caregiver-related determinants encompass dietary practices, oral hygiene behaviors, feeding patterns, and parental or caregiver influences that directly shape children's exposure to cariogenic conditions during early childhood. Second, social and contextual determinants include socioeconomic circumstances, educational factors, living environments, access to preventive resources, and healthcare system characteristics that influence both exposure to risk factors and opportunities for prevention. Third, biological and health-related determinants comprise microbial, anatomical, physiological, and general health factors that may modify individual susceptibility to ECC. Although presented separately for interpretative purposes, these determinant groups interact dynamically and should not be viewed as independent pathways. Rather, ECC appears to emerge from the cumulative influence of behavioral, social, biological, and environmental exposures operating across the early life course. This conceptual grouping provides a useful framework for understanding how different determinants contribute to ECC risk and for identifying potential targets for oral health promotion interventions.

### Behavioral and caregiver-related determinants

Behavioral and caregiver-related determinants emerged as the most consistently reported and potentially modifiable factors associated with ECC across the included reviews (14, 16, 18, 19). These determinants encompass dietary practices, oral hygiene behaviors, feeding routines, and caregiver influences that collectively shape children's oral health environments during the critical early years of life. Although discussed separately in many primary studies, the available evidence suggests that these factors operate through interconnected pathways and are often influenced by common social and environmental conditions. Consequently, ECC should not be viewed as the consequence of isolated behaviors but rather as the result of cumulative exposures occurring within the family and caregiving environment. This interpretation is supported by broader evidence demonstrating that ECC develops through complex interactions between behavioral practices, caregiver characteristics, biological susceptibility, and contextual influences (1, 3, 4, 20).

Dietary factors were among the most consistently reported determinants across the included reviews (14, 16, 18, 19). Frequent consumption of sugary foods and beverages, nocturnal feeding, feeding on demand, early introduction of sugar-sweetened products, and high household expenditure on sugary foods were repeatedly associated with increased ECC risk. These findings are biologically plausible given the established role of fermentable carbohydrates in the caries process. However, evidence from longitudinal studies suggests that dietary exposures rarely act independently and that their impact may be modified by oral hygiene practices, fluoride exposure, and previous disease experience (3, 21). In many African settings, ongoing nutritional transitions, increased availability of processed foods, and limited access to preventive resources may further amplify the effects of frequent sugar consumption. From an oral health promotion perspective, these findings support interventions aimed at reducing children's exposure to free sugars through caregiver education, nutrition counseling, and broader public health policies promoting healthier food environments (14, 18, 19).

Oral hygiene-related determinants were also consistently identified across reviews (14, 16, 18, 19). Infrequent toothbrushing, delayed initiation of oral hygiene practices, inadequate caregiver supervision, poor oral health knowledge, and increased plaque accumulation were repeatedly associated with ECC. Evidence from previous systematic reviews suggests that oral hygiene behaviors may exert their protective effects primarily through plaque control and fluoride delivery rather than through brushing frequency alone (1, 3, 22). Consequently, the effectiveness of oral hygiene practices depends not only on their frequency but also on caregiver involvement, oral health literacy, and access to fluoridated toothpaste. These findings highlight the importance of promoting early oral hygiene habits, strengthening caregiver support, and improving access to affordable preventive resources during early childhood.

Feeding practices and caregiver-related influences constituted another important group of determinants (14, 16, 18, 19). Prolonged breastfeeding, nocturnal feeding, bottle-feeding with sweetened contents, delayed weaning, and inappropriate feeding routines were frequently associated with ECC. At the same time, caregiver oral health knowledge, attitudes, supervision practices, and awareness of the importance of primary teeth were identified as factors influencing children's oral health outcomes (16, 19). Evidence from Ma et al. (4) further suggests that broader psychosocial factors, including parental mental health and caregiving capacity, may indirectly affect children's dietary habits, oral hygiene routines, and healthcare utilization. Taken together, these findings emphasize the central role of caregivers in shaping children's oral health behaviors and support the integration of oral health promotion into maternal and child health services. Interventions targeting caregivers may provide a particularly effective approach for improving oral health literacy, strengthening preventive behaviors, and reducing ECC burden during the early years of life (14, 19, 23).

### Social and contextual determinants of oral health inequalities

Beyond individual and caregiver-related behaviors, the findings of this umbrella review highlight the important contribution of social and contextual determinants to ECC risk across African settings. Sociodemographic disadvantage emerged as one of the most consistently reported determinants, with several reviews identifying associations between ECC and lower household income, lower parental educational attainment, unemployment, disadvantaged residential environments, and limited access to healthcare services (14, 16, 18, 19). These findings are consistent with the broader social determinants of health framework, which recognizes that health outcomes are strongly influenced by the social, economic, and environmental conditions in which individuals are born, grow, and live. Rather than acting as isolated risk factors, socioeconomic conditions shape children's exposure to multiple behavioral and environmental determinants simultaneously, thereby influencing oral health outcomes throughout early childhood.

The association between sociodemographic disadvantage and ECC has important implications for understanding oral health inequalities in African populations. Children from disadvantaged households are often exposed to less favorable dietary environments, reduced access to fluoridated oral hygiene products, lower levels of oral health literacy, and greater barriers to preventive healthcare services (14, 15, 18, 19). School-related indicators, residential location, and community-level deprivation were also identified as factors associated with ECC in some reviews, suggesting that social and environmental contexts may influence children's oral health beyond individual household characteristics (19, 24). These findings support growing evidence indicating that oral diseases disproportionately affect vulnerable and underserved populations and contribute to the persistence of health inequalities across the life course.

Contextual determinants related to healthcare systems and preventive resources were reported less frequently but remain highly relevant from a public health perspective (16, 18, 19). Factors such as fluoride exposure, fluoride supplementation, healthcare utilization patterns, previous dental visits, and distance to oral health facilities may influence both the development of ECC and opportunities for prevention. In many African settings, preventive oral healthcare services remain limited, and dental attendance is often treatment-oriented rather than prevention-oriented. Consequently, many children first encounter oral healthcare services only after disease progression has occurred. Similar concerns have been highlighted in previous reviews, which emphasized the importance of access to preventive services and fluoride-based interventions in reducing ECC burden (1, 3).

From an oral health promotion perspective, these findings suggest that effective ECC prevention requires action beyond individual behavior change alone. Strategies focused exclusively on caregiver education may have limited impact if broader social and structural barriers remain unaddressed. Reducing oral health inequalities therefore requires multilevel approaches that improve access to preventive oral healthcare services, increase the availability and affordability of fluoridated toothpaste, strengthen community-based prevention initiatives, and integrate oral health promotion within broader child health and social development programs (14–19). Such approaches are particularly important for vulnerable and underserved populations, who often experience the greatest disease burden while facing the greatest barriers to prevention and care. By addressing upstream social and contextual determinants, oral health promotion initiatives may contribute not only to reducing ECC prevalence but also to improving equity in child oral health outcomes across African settings.

### Biological and health-related determinants

Biological and health-related determinants were reported less frequently across the included reviews and were generally supported by a smaller body of evidence than behavioral, caregiver-related, and socioeconomic factors (16, 19). Reported biological determinants included dental plaque accumulation, cariogenic microorganisms such as *Streptococcus mutans*, salivary characteristics, tooth anatomy, and previous caries experience, whereas health-related determinants included HIV infection, low CD4 count, sickle cell anemia, underweight and overweight status, low BMI-for-age z-scores, and other indicators of compromised general health (16, 19, 20). These factors are biologically plausible and may increase susceptibility to ECC through alterations in oral microbial ecology, immune function, nutritional status, or host defense mechanisms.

However, the available evidence suggests that biological and health-related determinants rarely act independently. Previous systematic reviews have identified plaque accumulation, cariogenic bacterial colonization, and baseline caries experience among the strongest predictors of future caries development, but these factors are themselves influenced by dietary habits, oral hygiene practices, fluoride exposure, and caregiver behaviors (1, 3, 21, 24). Similarly, general health conditions may increase vulnerability to ECC while simultaneously reflecting broader social and healthcare inequalities that affect access to preventive resources and oral healthcare services. Consequently, biological susceptibility should be viewed as one component of a multifactorial disease process rather than as a separate causal pathway.

From an oral health promotion perspective, these findings reinforce the importance of integrating oral health within broader child health frameworks. Children affected by chronic medical conditions, nutritional disorders, hematological diseases, or immunocompromising conditions may benefit from targeted preventive interventions, early risk assessment, and closer collaboration between oral health professionals and other healthcare providers. Nevertheless, the certainty of evidence supporting biological and health-related determinants remained low, largely because these factors were reported in relatively few reviews and were characterized by substantial heterogeneity. Further longitudinal research is therefore needed to clarify the role of biological susceptibility and general health conditions in shaping ECC risk across diverse African populations (16, 19, 22).

### Implications for oral health promotion

The findings of this umbrella review have important implications for oral health promotion across African settings. The identified determinants indicate that ECC is not solely the result of individual behaviors but rather emerges from the interaction of behavioral, caregiver-related, social, contextual, biological, and health-related factors operating throughout early childhood. Consequently, prevention strategies should move beyond treatment-oriented approaches and adopt comprehensive oral health promotion frameworks that address both proximal determinants, such as feeding and oral hygiene practices, and upstream determinants, including sociodemographic disadvantage, limited access to preventive resources, and healthcare inequalities. Such an approach is consistent with contemporary oral health promotion models that emphasize prevention, equity, and the creation of supportive environments for health.

Caregiver-centered interventions should constitute a central component of ECC prevention strategies. Since many of the most consistently reported determinants are directly influenced by parental knowledge, attitudes, and daily caregiving practices, integrating oral health education into maternal and child health services may provide an effective mechanism for reaching families during critical developmental periods. Educational initiatives should promote appropriate feeding practices, reduction of free sugar consumption, early initiation of toothbrushing with fluoridated toothpaste, and timely utilization of preventive dental services. In addition, strengthening oral health literacy among caregivers may improve their capacity to adopt and sustain preventive behaviors that support healthy oral development during childhood.

Community-based oral health promotion programs may further contribute to reducing ECC burden and improving access to prevention. Delivery of preventive interventions through primary healthcare centers, preschools, schools, community organizations, and outreach programs may facilitate early engagement with families, particularly in underserved and rural populations. Such approaches can increase awareness of ECC risk factors, support healthy behavioral practices, and promote earlier identification of children at increased risk of disease. Community-based strategies may be particularly relevant in African settings where oral healthcare infrastructure remains unevenly distributed and access to preventive services is frequently limited.

The findings also support greater integration of oral health promotion within maternal and child health services and other primary healthcare programs. Pediatricians, nurses, midwives, nutritionists, community health workers, and oral health professionals all represent potential partners in ECC prevention. Incorporating oral health counseling, risk assessment, dietary guidance, and preventive advice into routine child health visits may increase the reach of preventive interventions while facilitating earlier identification of vulnerable children. Such interprofessional approaches may be especially valuable in resource-constrained settings where access to specialized oral healthcare services remains limited.

At the policy level, the evidence supports the implementation of population-based strategies aimed at creating healthier oral environments. Improving the availability and affordability of fluoridated toothpaste, supporting fluoride-based preventive programs where appropriate, strengthening preventive oral healthcare services, and promoting policies that reduce children's exposure to sugar-rich foods and beverages may contribute substantially to reducing ECC burden. Because sociodemographic disadvantage emerged as one of the most consistent determinants of disease, oral health policies should also prioritize vulnerable and underserved populations and seek to reduce structural barriers that limit access to prevention and care. Such efforts are aligned with broader public health objectives related to health equity, universal health coverage, and the reduction of health inequalities.

Emerging digital health technologies may provide additional opportunities to strengthen oral health promotion activities across African settings. Mobile health applications, telehealth platforms, text-message reminders, and digital educational resources may facilitate caregiver education, support behavior change, and improve dissemination of preventive information, particularly in areas with limited access to oral health professionals. Although evidence regarding their effectiveness for ECC prevention in African contexts remains limited, digital approaches may complement traditional community-based interventions and enhance the scalability of oral health promotion programs.

Overall, the evidence synthesized in this review supports a shift from predominantly treatment-oriented models toward comprehensive prevention and oral health promotion strategies. Addressing social determinants of health, strengthening caregiver support, improving access to preventive services, promoting interprofessional collaboration, and implementing population-level policies may collectively contribute to reducing ECC burden and narrowing oral health inequalities among African children. Future oral health promotion initiatives should incorporate robust monitoring and evaluation frameworks to assess effectiveness, scalability, sustainability, and equity of impact across diverse African settings.

The interpretation of these findings should consider the methodological characteristics of the included reviews. AMSTAR 2 assessments ranged from high to critically low quality, while ROBIS judgments indicated predominantly low overall risk of bias, with only two reviews classified as high risk. Furthermore, the narrative GRADE assessment supported moderate certainty for ECC prevalence and the most consistently reported determinant categories, whereas evidence supporting biological, health-related, maternal/caregiver-related, and contextual determinants remained limited. The low corrected covered area (CCA = 4.7%) further suggests minimal overlap between reviews and reduces concerns regarding redundancy of primary evidence.

## Strengths and limitations

This umbrella review has several important strengths while also presenting limitations that should be considered when interpreting the findings. A major strength lies in the comprehensive synthesis of evidence on ECC prevalence and determinants across African settings through the inclusion of systematic reviews and meta-analyses. By focusing specifically on ECC and applying clearly defined eligibility criteria, the review provides a focused overview of a major oral health challenge affecting young children across the continent. The use of a structured methodological approach, including a comprehensive search strategy, AMSTAR 2 appraisal, ROBIS assessment, and narrative GRADE evaluation, further strengthens the transparency and methodological rigor of the review. In addition, the assessment of overlap through a citation matrix and corrected covered area (CCA) calculation enabled evaluation of redundancy within the evidence base and provided additional insight into the independence of the included reviews.

Several limitations should also be acknowledged. The number of eligible systematic reviews remained limited, and important geographical gaps persist across several African regions. Considerable methodological heterogeneity was observed among the included reviews, including differences in diagnostic criteria, age ranges, study settings, sampling methods, and definitions of ECC-related exposures. Variability in methodological quality, ranging from high to critically low confidence according to AMSTAR 2, may also have influenced the robustness of some findings.

The narrative GRADE assessment indicated variability in the certainty of evidence across outcomes, with lower certainty observed for several determinant categories due to limitations in the available evidence base. Furthermore, although ROBIS assessments suggested generally acceptable methodological quality, some reviews presented concerns related to study identification procedures, appraisal methods, and synthesis approaches. The predominance of cross-sectional studies within the primary evidence base further limits causal inference and restricts interpretation of many reported associations.

The assessment of overlap demonstrated a low corrected covered area (CCA = 4.7%), indicating minimal redundancy among included reviews. While this reduces the risk of double-counting and supports the breadth of evidence included in the synthesis, it may also contribute to variability in findings because conclusions are derived from largely distinct primary studies. The highest overlap was observed between the two Nigerian reviews, whereas overlap across reviews conducted in different African regions remained limited.

Furthermore, several determinant categories were informed by a limited number of systematic reviews, particularly biological, maternal/caregiver-related, health-related, and contextual determinants. This limited evidence base may reduce the stability, precision, and generalizability of some findings. Consequently, determinants supported by fewer reviews should be interpreted with caution until additional high-quality evidence becomes available across diverse African settings.

The findings should also be interpreted within the geographical scope of the review. By restricting inclusion to African populations, this umbrella review provides context-specific evidence relevant to African oral health systems and oral health promotion strategies. However, the findings may not be fully generalizable to other world regions with different socioeconomic, cultural, healthcare, and epidemiological contexts. Future umbrella reviews incorporating evidence from multiple geographic regions may help identify both common and context-specific determinants of ECC and strengthen the global applicability of prevention strategies.

Finally, the restriction to English-language publications may have resulted in the exclusion of relevant evidence published in other languages and should be considered when interpreting the findings.

## Conclusion

This umbrella review provides a comprehensive synthesis of the available evidence regarding the prevalence and determinants of early childhood caries (ECC) across African settings. The findings indicate that ECC remains a substantial oral health burden among young children, with considerable variation across regions and a multifactorial etiology involving behavioral, sociodemographic, maternal/caregiver-related, biological, health-related, and contextual determinants. Dietary practices, oral hygiene behaviors, sociodemographic determinants, and breastfeeding and bottle-feeding-related factors emerged as the most consistently reported determinants and may therefore represent priority targets for prevention and intervention.

The evidence supports the implementation of comprehensive oral health promotion strategies that strengthen caregiver education, improve access to preventive oral healthcare services, promote appropriate fluoride exposure, and integrate oral health into maternal and child health programs. These findings reinforce the need for integrated oral health promotion approaches that address both individual behaviors and the broader social determinants of child oral health. Effective prevention of ECC requires coordinated action across healthcare, educational, community, and policy sectors to address the multiple determinants influencing children's oral health.

Particular attention should be directed toward vulnerable and underserved populations, who often experience a disproportionate burden of disease and reduced access to preventive resources. Community-based interventions, interprofessional collaboration, integration of oral health within nutrition and child health services, and population-level policies aimed at creating healthier environments may contribute substantially to reducing ECC burden and oral health inequalities across African settings.

Although methodological heterogeneity remains, the available evidence supports the development, implementation, and evaluation of context-specific prevention policies and oral health promotion programs tailored to the needs of African children and their caregivers. Future research should prioritize the evaluation of oral health promotion interventions, the investigation of contextual and health system determinants, and the assessment of integrated child health approaches to strengthen the evidence base for equitable and sustainable ECC prevention across Africa.

## Data Availability

The original contributions presented in the study are included in the article/Supplementary Material, further inquiries can be directed to the corresponding author.
