# Supplementary material for: Early childhood caries in Africa: prevalence, determinants, and implications for oral health promotion—an umbrella review
Source: Front Oral Health. 2026 Jun 25;7:1901145. doi: 10.3389/froh.2026.1901145 (PMC13346219; doi:10.3389/froh.2026.1901145)
Supplement: Supplementary file 1 [file Table1.docx]

# Supplementary Appendix 1

## Full Electronic Search Strategies

## 1. MEDLINE (via PubMed)

(("early childhood caries"[Title/Abstract] OR "ECC"[Title/Abstract] OR "dental caries"[Title/Abstract] OR "tooth decay"[Title/Abstract] OR "caries"[Title/Abstract])AND ("prevalence"[Title/Abstract] OR"epidemiology"[Title/Abstract]
OR "risk factors"[Title/Abstract] OR "determinants"[Title/Abstract])
AND
("child"[Title/Abstract] OR "children"[Title/Abstract]
OR "preschool"[Title/Abstract] OR "preschool child"[MeSH Terms])
AND
("Africa"[Title/Abstract] OR "African"[Title/Abstract]
OR "Algeria"[Title/Abstract]
OR "Angola"[Title/Abstract]
OR "Benin"[Title/Abstract]
OR "Botswana"[Title/Abstract]
OR "Burkina Faso"[Title/Abstract]
OR "Burundi"[Title/Abstract]
OR "Cameroon"[Title/Abstract]
OR "Cape Verde"[Title/Abstract]
OR "Central African Republic"[Title/Abstract]
OR "Chad"[Title/Abstract]
OR "Comoros"[Title/Abstract]
OR "Congo"[Title/Abstract]
OR "Democratic Republic of Congo"[Title/Abstract]
OR "Djibouti"[Title/Abstract]
OR "Egypt"[Title/Abstract]
OR "Equatorial Guinea"[Title/Abstract]
OR "Eritrea"[Title/Abstract]
OR "Eswatini"[Title/Abstract]
OR "Ethiopia"[Title/Abstract]
OR "Gabon"[Title/Abstract]
OR "Gambia"[Title/Abstract]
OR "Ghana"[Title/Abstract]
OR "Guinea"[Title/Abstract]
OR "Guinea-Bissau"[Title/Abstract]
OR "Ivory Coast"[Title/Abstract]
OR "Côte d’Ivoire"[Title/Abstract]
OR "Kenya"[Title/Abstract]
OR "Lesotho"[Title/Abstract]
OR "Liberia"[Title/Abstract]
OR "Libya"[Title/Abstract]
OR "Madagascar"[Title/Abstract]
OR "Malawi"[Title/Abstract]
OR "Mali"[Title/Abstract]
OR "Mauritania"[Title/Abstract]
OR "Mauritius"[Title/Abstract]
OR "Morocco"[Title/Abstract]
OR "Mozambique"[Title/Abstract]
OR "Namibia"[Title/Abstract]
OR "Niger"[Title/Abstract]
OR "Nigeria"[Title/Abstract]
OR "Rwanda"[Title/Abstract]
OR "Sao Tome and Principe"[Title/Abstract]
OR "Senegal"[Title/Abstract]
OR "Seychelles"[Title/Abstract]
OR "Sierra Leone"[Title/Abstract]
OR "Somalia"[Title/Abstract]
OR "South Africa"[Title/Abstract]
OR "South Sudan"[Title/Abstract]
OR "Sudan"[Title/Abstract]
OR "Tanzania"[Title/Abstract]
OR "Togo"[Title/Abstract]
OR "Tunisia"[Title/Abstract]
OR "Uganda"[Title/Abstract]
OR "Zambia"[Title/Abstract]
OR "Zimbabwe"[Title/Abstract]))
AND
(systematic[sb] OR "systematic review"[Title/Abstract]
OR "meta-analysis"[Publication Type])
Filters: English language; Publication date from January 2000 to April 2026.

## 2. Scopus

TITLE-ABS-KEY(
("early childhood caries" OR ECC OR "dental caries" OR "tooth decay" OR caries)
AND
(prevalence OR epidemiology OR "risk factors" OR determinants)
AND
(child OR children OR preschool)
AND
(Africa OR African OR Algeria OR Angola OR Benin OR Botswana OR Burkina Faso
OR Burundi OR Cameroon OR "Cape Verde" OR Chad OR Comoros OR Congo
OR "Democratic Republic of Congo" OR Djibouti OR Egypt OR Ethiopia
OR Gabon OR Gambia OR Ghana OR Guinea OR Kenya OR Lesotho OR Liberia
OR Libya OR Madagascar OR Malawi OR Mali OR Mauritania OR Mauritius
OR Morocco OR Mozambique OR Namibia OR Niger OR Nigeria OR Rwanda
OR Senegal OR Seychelles OR "Sierra Leone" OR Somalia OR "South Africa"
OR Sudan OR Tanzania OR Togo OR Tunisia OR Uganda OR Zambia OR Zimbabwe)
)
AND
(TITLE-ABS-KEY("systematic review") OR TITLE-ABS-KEY("meta-analysis"))
AND PUBYEAR > 1999
AND PUBYEAR < 2027
AND (LIMIT-TO(LANGUAGE, "English"))

## 3. Web of Science

TS=(
("early childhood caries" OR ECC OR "dental caries" OR "tooth decay" OR caries)
AND
(prevalence OR epidemiology OR "risk factors" OR determinants)
AND
(child OR children OR preschool)
AND
(Africa OR African OR Algeria OR Angola OR Benin OR Botswana OR Burkina Faso
OR Burundi OR Cameroon OR Chad OR Comoros OR Congo
OR "Democratic Republic of Congo" OR Djibouti OR Egypt OR Ethiopia
OR Gabon OR Gambia OR Ghana OR Guinea OR Kenya OR Lesotho OR Liberia
OR Libya OR Madagascar OR Malawi OR Mali OR Mauritania OR Mauritius
OR Morocco OR Mozambique OR Namibia OR Niger OR Nigeria OR Rwanda
OR Senegal OR Seychelles OR "Sierra Leone" OR Somalia OR "South Africa"
OR Sudan OR Tanzania OR Togo OR Tunisia OR Uganda OR Zambia OR Zimbabwe)
AND
("systematic review" OR "meta-analysis")
)

Refined by:
LANGUAGES: (ENGLISH)
Timespan: 2000–2026
Indexes: SCI-EXPANDED, SSCI, ESCI

## 4. Embase

('early childhood caries':ti,ab OR ECC:ti,ab
OR 'dental caries':ti,ab OR 'tooth decay':ti,ab)
AND
(prevalence:ti,ab OR epidemiology:ti,ab
OR 'risk factors':ti,ab OR determinants:ti,ab)
AND
(child:ti,ab OR children:ti,ab OR preschool:ti,ab)
AND
(Africa:ti,ab OR African:ti,ab OR Algeria:ti,ab OR Angola:ti,ab
OR Benin:ti,ab OR Botswana:ti,ab OR Burkina Faso:ti,ab
OR Burundi:ti,ab OR Cameroon:ti,ab OR Chad:ti,ab
OR Congo:ti,ab OR 'Democratic Republic of Congo':ti,ab
OR Djibouti:ti,ab OR Egypt:ti,ab OR Ethiopia:ti,ab
OR Gabon:ti,ab OR Gambia:ti,ab OR Ghana:ti,ab
OR Guinea:ti,ab OR Kenya:ti,ab OR Lesotho:ti,ab
OR Liberia:ti,ab OR Libya:ti,ab OR Madagascar:ti,ab
OR Malawi:ti,ab OR Mali:ti,ab OR Mauritania:ti,ab
OR Mauritius:ti,ab OR Morocco:ti,ab OR Mozambique:ti,ab
OR Namibia:ti,ab OR Niger:ti,ab OR Nigeria:ti,ab
OR Rwanda:ti,ab OR Senegal:ti,ab OR Seychelles:ti,ab
OR 'Sierra Leone':ti,ab OR Somalia:ti,ab
OR 'South Africa':ti,ab OR Sudan:ti,ab
OR Tanzania:ti,ab OR Togo:ti,ab OR Tunisia:ti,ab
OR Uganda:ti,ab OR Zambia:ti,ab OR Zimbabwe:ti,ab)
AND
('systematic review'/de OR 'meta analysis'/de)

Limits:
English language;
Publication years 2000–2026.
